# COVID-19 autopsy reports from the Ga-East Municipal and the 37 Military Hospitals in Accra, Ghana

**DOI:** 10.4314/gmj.v54i4s.9

**Published:** 2020-12

**Authors:** Seth Attoh, Roxana P Segborwotso, Samuel K Akoriyea, Gina Teddy, Lawrence Edusei, Frederick Hobenu, Kwasi Agyemang-Bediako, Alfred Toppar, Raymond D Fatchu, Patrick K Akakpo

**Affiliations:** 1 Pathology Division, 37 Military Hospital, Accra, Ghana; 2 Medical Division, 37 Military Hospital, Accra, Ghana; 3 Ga-East Municipal Hospital, Accra, Ghana; 4 Institutional Care Division, Ghana Health Service, Accra, Ghana; 5 Ghana Centre for Health Systems and Policy Research, Ghana Institute of Management and Public Administration, Accra, Ghana; 6 Department of Pathology, Korle-Bu Teaching Hospital, Accra, Ghana; 7 Department of Pathology, School of Medical Sciences, University of Cape Coast and Cape Coast Teaching Hospital, Cape Coast, Ghana

**Keywords:** COVID-19, Ghana, autopsy, thromboemboli, Diffuse Alveolar Damage

## Abstract

**Introduction:**

Since the declaration of COVID-19 by the World Health Organisation (WHO) as a global pandemic on 11th March 2020, the number of deaths continue to increase worldwide. Reports on its pathologic manifestations have been published with very few from the Sub-Saharan African region. This article reports autopsies on COVID-19 patients from the Ga-East and the 37 Military Hospitals to provide pathological evidence for better understanding of COVID-19 in Ghana.

**Methods:**

Under conditions required for carrying out autopsies on bodies infected with category three infectious agents, with few modifications, complete autopsies were performed on twenty patients with ante-mortem and/or postmortem RT -PCR confirmed positive COVID-19 results, between April and June, 2020.

**Results:**

There were equal proportion of males and females. Thirteen (65%) of the patients were 55years or older with the same percentage (65%) having Type II diabetes and/or hypertension. The most significant pathological feature found at autopsy was diffuse alveolar damage. Seventy per cent (14/20) had associated thromboemboli in the lungs, kidneys and the heart. Forty per cent (6/15) of the patients that had negative results for COVID-19 by the nasopharyngeal swab test before death had positive results during postmortem using bronchopulmonary specimen. At autopsy all patients were identified to have pre-existing medical conditions.

**Conclusion:**

Diffuse alveolar damage was a key pathological feature of deaths caused by COVID-19 in all cases studied with hypertension and diabetes mellitus being major risk factors. Individuals without co-morbidities were less likely to die or suffer severe disease from SARS-CoV-2

**Funding:**

None declared

## Introduction

Coronavirus disease 2019 (COVID-19) was declared by the World Health Organization (WHO) on the 11^th^ of March 2020 as a global pandemic.^1^ COVID-19 is caused by Severe Acute Respiratory Syndrome-Corona Virus-2 (SARSCoV-2) an RNA virus that belongs to the Coronavirus family.

Other members of this family have been responsible for previous deadly outbreaks of infection.^2^ SARS-CoV-2, the virus responsible for the current outbreak causes severe disease in people with other co-morbid conditions such as diabetes mellitus and hypertension and also in older people. Majority of infected patients have however been reported to be asymptomatic.^3^

Though the number of deaths from COVID-19 have continued to increase worldwide, only a handful of reports on the pathologic manifestations of COVID-19 have been published as at April 2020.^2^ In Ghana, the total number of confirmed cases as of 14 August 2020 was 42,532 with 231 deaths, yet a report of pathological manifestations of COVID-19 is yet to be published from Ghana.^4^

In elderly people and patients with co-morbidities, the more severe clinical presentation and course of COVID-19 infection is characterized by the clinical picture of Acute Respiratory Distress Syndrome (ARDS) with its pathological correlate of Diffuse Alveolar Damage (DAD) and Hyaline Membrane (HM) formation in the alveoli.^2^,^5^ This pathological feature has been consistently reported in pathological descriptions of COVID-19 and appears to be the most significant cause of mortality and morbidity.^6^ Less commonly, pulmonary edema, fibrinous exudates, focal reactive type II pneumocyte hyperplasia and bronchopneumonia with scattered multinucleated giant cells have also been reported.^7^,^8^,^9^ Together, these pathological findings have guided management protocols around the world.

Pathological changes in other organs have not gained as much attention as those in the lungs probably because of the more overt clinical course of the lung pathology (ARDS). Pathological findings in the gastrointestinal tract have been reported to include segmental dilatation and stenosis of the small intestine.^7^ Other gastrointestinal findings include degeneration, necrosis and shedding of the gastrointestinal mucosa, occasional lymphocytic infiltration in the oesophageal squamous epithelium, and abundant infiltrating plasma cells and lymphocytes with interstitial oedema in the lamina propria of the stomach, duodenum and rectum.^7^ In the urinary system, there have been reports of non-specific severe acute tubular injury with endothelial injury pattern and evidence of direct parenchymal tubular epithelial and podocyte viral infection.^12^ Again, the presence of pre-existing cardiovascular disease, appears to predispose patients to the development of cardiovascular complications, which are in turn associated with higher mortality rates.^13^ As an underlying pathological mechanism, endotheliitis has been highlighted as a basis for the vascular manifestations of COVID-19 infection in all affected organs.^14^

Our objective was to present the histopathological findings following autopsy of 20 COVID-19 patients at the Ga-East Municipal and the 37 Military Hospitals in Accra, Ghana. We provide pathological evidence for better understanding and improved management of COVID-19 patients in Ghana.

## Methods

Ethical clearance with reference number 37MH-IRB IPN/MAST/402/2020 was received from the Institutional Review Board of the 37 Military Hospital. With few modifications to conditions required for carrying out autopsies on bodies infected with category three infectious agents, complete autopsies were performed between April 2020 and June, 2020, on twenty patients with either an RT-PCR confirmed positive COVID-19 results, or presumed cases but with a positive RT-PCR at autopsy. Post-mortems were performed (on average 2 days after death) following WHO guidelines for Infection Prevention and Control for the safe management of a dead body in the context of COVID-19.^16^ The structural design of suites used lacked negative pressure systems with filters. As a result, extractors were fitted to provide unidirectional airflow away from the Anatomical Pathology Team (APT) into the atmosphere as an appropriate technology. Also, each member of the APT was carefully selected to exclude those with chronic conditions such as diabetes mellitus, cardiovascular and respiratory diseases. The clinical data of each patient was reviewed prior to the autopsy. The autopsy examination included complete gross external and internal examinations in addition to histopathological examination of all major wellpreserved organs (lungs, heart, liver, kidney, brain).

## Results

Twenty patients of age between 20 years and 79 years who tested positive for COVID-19 prior to or after death are presented. There was equal proportion of males and females. Thirteen (65%) of our patients were 55years or older with the same percentage (65%) having Type II Diabetes Mellitus (DM) and/or hypertension (HPT). Most of these patients presented with respiratory symptoms of varying severity with decreased saturation on intranasal oxygen (INO^2^) in association with other constitutional symptoms such as fever, headache, malaise, generalized weakness and collapse. There was an out of the ordinary patient (Case17) who had an initial presentation of tonic clonic seizures with epistaxis, haematuria and bleeding from the vagina with decreased platelets (46 × 10^9^/L). She later developed respiratory distress. Almost all the patients had co-morbid conditions. Details of patients' clinical presentation are summarized in Table 1

**Table 1:** Clinical summary of 20 cases with ARDS and positive RT-PCR at autopsy

ID	Sex	Age (yr)	Presentation	Clinical Signs	Co-morbidities	Results of Imaging	Results of Lab investigations	Clinical diagnosis and Treatment	Significant autopsy findings	Cause of Death
1.	M	41	Cough, difficulty in breathing, headache for five days, positive travel history	Obese and Cyanosed	DM, obesity	Patchy opacification on chest X-ray	Elevated blood glucose COVID-19 positive	HPT/DKA Received insulin / antibiotics, INO_2_ Died four days after admission	Severe congestion and oedema of lungs with foci of consolidation. Thrombus in coronary vessel	COVID-19 pneumonia with severe DM
2.	M	55	Fever, difficulty in breathing, sore throat, chest pain for a week	Fever, reduced oxygen saturation	HPT/DM	Ground-glass appearance on chest X-ray	Elevated blood glucose and COVID-19 positive	HPT/DM Received Azithromycin, Hydroxychloroquine, Metformin and Glibenclamide. Died three days after admission	Central cyanosis, Haemorrhage on pleural surfaces, massive right PE	Acute PE from COVID-19 pneumonia in a HPT/DM.
3.	F	39	Generalized weakness, difficulty in breathing for two weeks.	Respiratory distress, bronchial breath sounds with bilateral basal crepitations	HIV	Patchy opacification on chest X-ray	HIV I and II Positive COVID-19 Positive.	Bilateral pneumonia, COVID-19. Started on Ceftriaxone, Azithromycin, Prednisolone, Dalteparin and Co-trimoxazole. Died four days after admission	Advanced Autolysis of internal organs. Congestion and oedema Interstitial fibrosis, eosinophilic masses in alveoli	COVID-19 pneumonia in a patient with HIV
4.	F	33	Severe headache, dizziness, palpitations, blocked nostrils, difficulty in breathing, fever, vomiting for a month	High BP	Late Pregnancy	Live Fetus on USG, diffuse bilateral hilar opacity on X-ray	D-dimers elevated COVID-19 positive	PE, COVID-19 pneumonia. Pre-eclampsia. Received Heparin, Amoxicillin clavulanate, Azithromycin, MgSO4, Methyl dopa, Dexamethasone, INO_2_ Died after four days	Congested Lungs, LVH, subcapsular liver hemorrhage	COVID-19 pneumonia in a patient with pre-eclampsia
5.	F	66	Right-sided weakness, aphasia, difficulty in breathing, headache for four days	Respiratory distress, dull percussion note, bronchial breath sounds	HPT	Head CT showed cystic dilatation of left frontal lobe, small vessel ischaemia	COVID-19 positive	PE, COVID-19. Dalteparin, Azithromycin, Zinc and Vitamin C. Died same day	Obese, petechial hemorrhage on pleural surfaces, congestion and edema of lungs, PE	PE, COVID- 19 in a hypertensive with small vessel ischemia
6.	M	60	Cough, dyspnea, palpitations for a week	Unconscious, diaphoretic, reduced air entry bilaterally	HPT/DM	Nil	Low platelet count, Elevated D-dimer No COVID-19 test done	Bilateral Pneumonia, MI On Metformin and Amlodipine Died same day	Cyanosed, petechial hemorrhages on pleural surfaces, severe congestion, moderate edema, Bilateral Massive PE, CLVH	Bilateral Massive PE from COVID-19 pneumonia in a known HPT/DM
7.	F	79	General malaise, difficulty in breathing, left leg pain for two weeks.	Febrile, respiratory distress, reduced air entry, bronchial breath sounds	Chronic kidney disease	Patchy opacification on chest X-ray	Anemia, Elevated potassium, creatinine, and urea. Low EGFR COVID-19 positive	Pneumonia, chronic kidney disease Given Azithromycin, Dalteparin, Hydroxychloroquine, Zinc and Vitamin C Died same day	Cyanosis, congested, firm and mildly edematous lungs, shrunken kidneys, coarsely granular sub-capsular surfaces, poor cortico-medullary differentiation. Microthrombi in pulmonary arteries	COVID-19 bronchopneumonia in a patient with chronic kidney disease
8.	M	70	Anorexia, lethargy for three days	Pale, febrile, transmitted sounds coarse crepitations.	HPT/DM/ Bronchitis	Patchy opacification on chest X-ray	Elevated blood glucose, COVID-19 positive	COVID-19, HPT/DM. Given Insulin, Azithromycin, Ceftriazone, Dalteparin Died after two days on admission	Cyanosis Bilateral pleural effusion, Bilateral patchy consolidation, LVH	COVID-19 bronchopneumonia in a known HPT/DM with Bronchitis
9.	F	70	Difficulty in breathing, weakness, lethargy for two weeks	Respiratory distress, Reduced air entry bilaterally, morbid obesity	HPT/DM, obesity	Ground glass appearance on chest X-ray	Elevated blood glucose, COVID-19 positive	HPT/DM, COVID-19 Azithromycin, Ceftriazone, Dalteparin, Hydrocortisone Died same day	Morbidly obese, central cyanosis, Edematous and congested lungs, Bilateral hypostatic consolidation, Bilateral PE.	Bilateral PE, COVID-19 pneumonia in a known HPT/DM
10.	F	60	Difficulty in breathing, cough for three weeks.	Obese, respiratory distress	HPT/DM, obesity	Chest CT scan showed consolidation with air bronchograms	High D-dimer, elevated WBC, Deranged liver enzymes COVID-19 negative	PE, CCF, COVID-19 Given Azithromycin, Ceftriazone, Dalteparin, Hydrocortisone, Insulin and Amlodipine Died after 10 days of admission	Petechial hemorrhages on pleural surfaces heavy lungs with severe congestion and edema. PE, CLVH	Acute PE due to COVID-19 Pneumonia in a HPT/DM
11.	M	55	Cough, dyspnea, fever, general weakness, headache for seven days. Positive travel history	Respiratory distress, febrile, bronchial breath sounds, basal crepitations	HPT/DM	X-ray and CT scan showed ground glass appearance	Elevated blood glucose, Low Hb, High WBC No COVID-19 test done	Bilateral COVID-19 pneumonia, HPT/DM Given Azithromycin, Ceftriazone, Dalteparin, Hydrocortisone, Insulin and Amlodipine Died after two days of admission	Petechial hemorrhages on lung surface. Heavy lungs with severe congestion and moderate edema. Bilateral massive PE.	PE, COVID-19 pneumonia with HPT/DM
12.	F	37	Difficulty in breathing, bilateral pedal swelling, abdominal distention for two weeks	Cyanosed, mild jaundice, bi-pedal pitting edema, fever, diastolic murmur	Atrial myxoma Post-thyroidectomy, CCF	Echocardiography-Atrial myxoma-	COVID-19 negative	CCF secondary to atrial myxoma, bilateral pneumonia. Started on Furosemide, azithromycin, Ceftriaxone, Fluconazole, Aldactone, Thyroxine Died after three days of admission	Severely congested lungs with bilateral hypostatic consolidation, atrial myxoma with thrombus, CCF, MI	COVID-19 pneumonia, CCF due to left atrial myxoma
13.	M	62	General malaise, easy fatigability, difficulty in breathing for two weeks	Respiratory distress, Reduced air entry bilaterally with crepitations	HPT	CT scan of the chest showed extensive peripheral and basal ground glass opacities	Elevated blood sugar, COVID-19 negative	Bilateral pneumonia, COVID-19, HPT/DM Given Azithromycin, Ceftriazone, Dalteparin, Hydrocortisone, Insulin and Amlodipine Died six days after admission	Both lungs are heavy with severe congestion and moderate edema. Bilateral hypostatic consolidation	COVID-19 bronchopneumonia in a HPT/DM
14.	M	62	Fever, difficulty in breathing, cough for five days	Fever, reduced oxygen saturation, reduced air entry with bronchial breath sounds	HPT	Nil	COVID-19 test not done	Acute left ventricular failure due to HPT, COVID-19, PE Given furosemide, Enoxaparin Died in ambulance	Congested, edematous lungs, dilated left ventricle with LVH, MI Microthrombi in pulmonary arteries	COVID-19 pneumonia. Acute left ventricular failure due to MI in a known hypertensive
15.	M	61	Generalized weakness, difficulty in breathing, fever, dry cough for six hours	Respiratory distress, fever, basal crepitations bilaterally, reduced SPO_2_	HPT/DM	Nil	Elevated blood sugar No COVID-19 test done	Bilateral Pneumonia, COVID-19. Started on Ceftriaxone, Azithromycin, Dalteparin, Co-trimoxazole. Died following day	Heavy congested lungs, CLVH	COVID-19 pneumonia in a known HPT/DM
16.	M	57	Found dead in room	Nil	HPT	Nil	No COVID-19 test done	Unknown Brought-in-dead	Right lower lobe consolidation, CLVH, Microthrombi in pulmonary vessels	COVID-19 pneumonia with secondary bacterial infection in a known hypertensive
17.	F	20	Fever, vomiting, diarrhea for three days, sudden collapse	Respiratory distress, unrecordable BP, high pulse rate, tonic clonic seizures, haematuria, epistaxis, vaginal bleeding	None	Nil	Elevated blood sugar, low platelet, Retro negative, pregnancy test negative COVID-19 Positive	Shock, DIC, Gastroenteritis Started on Dexamethazone, Clindamycin, Insulin, Phenytoin, Meropenem, Fresh frozen Plasma Died same day	Cyanosed, Severe systemic congestion Congested and edematous lungs Microthrombi in pulmonary vessels	COVID-19 pneumonia in a newly diagnosed diabetic
18.	F	31	Increasing difficulty in breathing, cough for a week	Fever, contractions,	35 weeks Pregnant, one previous C/S	Patchy opacification on chest X-ray	COVID-19 Negative	Atypical Pneumonia, COVID-19, Emergency C/S after 2 days, Azithromycin, Cefuroxime, Dalteparin, Zinc, Vitamin C, Dexamethazone Died 6 days after C/S	Congested and heavy lungs	COVID-19 pneumonia in pregnancy
19.	F	28	Worsening breathlessness for four days	Fever, reduced air entry bilaterally, low SPO_2_	None	Ground-glass appearance on chest X-ray	Thrombocytopaenia COVID-19 Negative	COVID-19 pneumonia to rule out PE. Given Dalteparin, Ceftriaxone, Azithromycin and Dexamethasone Died same day	Systemic Congestion, Firm congested Lungs, Left Adrenal Adenoma, Microthrombi in lungs and kidneys	COVID-19 pneumonia with adrenal adenoma
20.	M	61	Sore throat, difficulty in breathing, general body weakness, diarrhea, cough for four days, right leg ulcer	Obese, pale, fever, swollen, warm and ulcerating right leg	HPT/DM	Patchy opacification on chest X-ray	COVID-19 negative	Diabetes Mellitus with Right leg ulcer, MI, PE. Given Ceftriazone, Clindamycin Dalteparin, Azithromycin, Insulin, Amlodipine, Acetyl salicylic acid Died following day	Congested, firm, heavy lungs, fibrinous exudates on lung surfaces, PE, CLVH	Massive PE with COVID-19 pneumonia in a known HPT/DM

Grossly, the significant autopsy findings were respiratory in nature. Findings included, pulmonary thromboemboli (PE) involving both the larger and smaller pulmonary artery branches of the lungs in some patients (details provided in Table 1).

This was associated with petechial haemorrhage on the surfaces of the lungs and areas of hemorrhagic infarction. In general, however there were frothy secretions in the larynx, trachea and bronchi, and the lungs were usually heavy, firm, severely congested with moderate to severe pulmonary edema and various degrees of consolidation. The hearts of hypertensive patients were enlarged and showed concentric left ventricular hypertrophy (CLVH) in keeping with hypertensive heart disease. There was an isolated case of a patient with an atrial myxoma (Case 12) who had a friable soft gelatinous mass measuring 45x30mm attached to the anterior wall of the left atrium, 12mm above the annulus of the anterior leaflet of the mitral valve. This was consistent with the antemortem clinical diagnosis. Again, this patient had isolated dilatation of all the chambers of the heart except the left ventricular chamber. In this patient and another (Case 14), there were areas of scaring in the left ventricle suggestive of previous myocardial infarction.

The liver was heavy and showed fatty change in most patients. In general, however the liver was moderate to severely congested in all patients though no other significant pathology was found except in a pregnant patient who had a subcapsular bleed (Case 4).

The kidneys showed no significant changes in most patients except one in whom the kidneys were small in size and showed coarsely granular subcapsular surface (Case 7). There was a patient with an adrenal adenoma (Case 19) and another with a cystic mass in the brain (Case 5) but no other patient had any significant findings in the brain. There were no other significant findings in any other organs.

**Figure 1:**
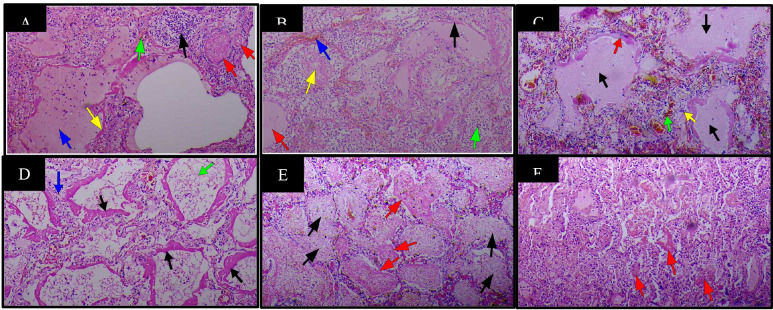
Haematoxylin & Eosin staining of lung tissue samples in patients with COVID-19(37 Military Hospital, Accra, Ghana, April 2020 to June 2020) X100. **A**. Diffuse alveolar damage (DAD) with presence of hyaline membrane (HM) (yellow arrow), alveolar oedema (blue arrow), congestion of pulmonary capillary (green arrow), focal accumulation of neutrophils (black arrow) and microthrombi (red arrows). **B**. DAD with HM (black arrow), oedema (red arrow), congestion (blue arrow), intra-alveolar accumulation of neutrophils (green arrow) and eosinophilic blobs(yellow arrow). **C**. DAD with HM (red arrow), oedema (black arrows), congestion (green arrow) with peri-vascular lymphocytic infiltrates (yellow arrow). **D**. DAD with HM formation in a moderately preserved body (black arrows), oedema (green arrow) and interstitial fibrosis with lymphocytic infiltrate (blue arrow). **E.** Alveolar exudate consisting of neutrophils, red blood cells, macrophages (black arrows) and poorly formed HM (red arrows). **F**. HM appearing as eosinophilic blobs in a poorly preserved body (red arrows)

**Figure 2:**
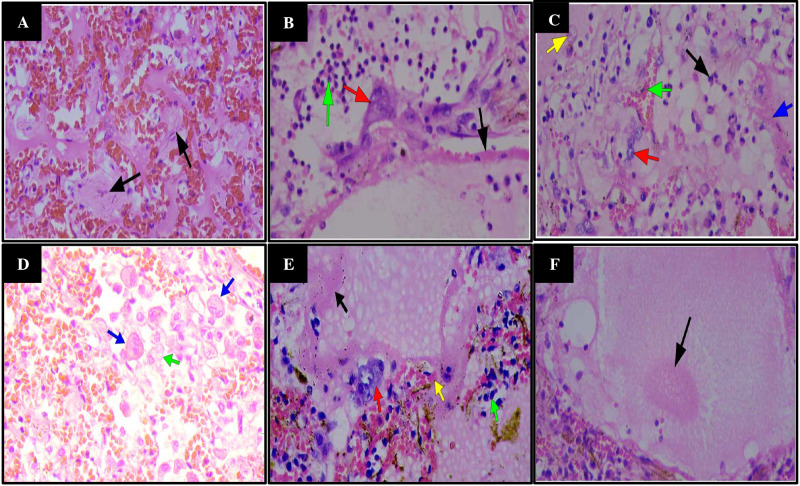
Hematoxylin & Eosin staining of lung tissue samples of patients with COVID-19 (37 Military Hospital, Accra, Ghana, April 2020 to June 2020) X400. **A**. Severe congestion with intra-alveolar proliferation of fibroblasts (black arrows). **B**. Neutrophilic accumulation (green arrow), Type 2 pneumocyte hyperplasia (red arrow) and HM presence (black arrow). **C**. Macrophage (yellow arrow), congestion (green arrow), Type 2 pneumocytes (red arrow), neutrophils (black arrow) and HM (blue arrow). **D**. Intra-alveolar macrophages (green arrow) including multinucleated giant cells (blue arrows). **E**. DAD with HM formation (black arrow), Type 2 pneumocyte hyperplasia (red arrow), congestion with peri-vascular lymphocytic infiltrates (green arrow). **F**. Formation of eosinophilic blob in an alveolus

**Figure 3:**
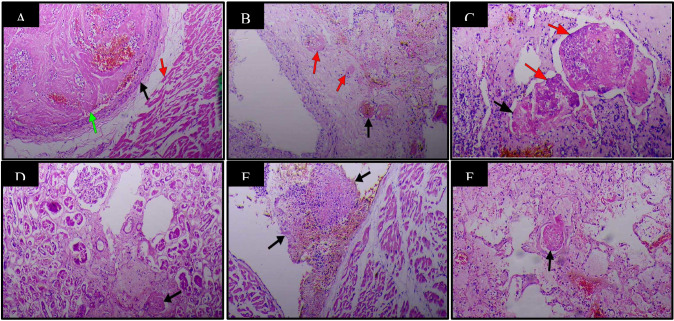
Haematology & Eosin staining of various organs and tissues with microthrombi in patients with COVID-19 (37 Military Hospital, Accra, Ghana, April 2020 to June 2020) X100. **A**. Section of the heart muscle (red arrow) showing a thrombus (green arrow) in a coronary artery (black arrow). **B**. Vasa vasora (arrows) of a branch of pulmonary artery showing thrombi (red arrows). **C**. Thrombi(arrows) in a vessel of an atrial myxoma with presence of vegetations (red arrows). **D**. Thrombus in an arteriole of the kidney(arrow). **E**. Thrombus on the surface of the heart(arrows). **F**. Thrombus in a pulmonary arteriole(arrow)

The most significant findings on histopathological examination were respiratory, in agreement with the gross findings. Sections from patients' lungs showed severe edema within the lungs (Figure 1A, B, C, D), sometimes seen as eosinophilic blobs (Figure 1F& 2 F), with mild to severe inflammatory cell exudates, made up of neutrophils, lymphocytes, macrophages, giant cells with a predominance of different cells in different patients (Figure 1A&E, 2B, 2D). There are fibroblasts in areas (Figure 2A). In some patients, lymphocytes dominated while in others, neutrophils predominated (Case 8) (Figure 1B). There were scattered macrophages including multinucleated giant cells (Cases 1,2,9 and 10) (Figure 2D). These inflammatory cells were in the alveolar spaces and the interstitium. There were prominent atypical pneumocytes with prominent type II pneumocyte proliferation in most patients (Figure 2. B, C, D, E). These pneumocytes were large and showed enlarged nuclei and granular amphophilic cytoplasm (Figure 2 B). The most significant finding was DAD and widespread HM seen lining the alveoli in all cases (Figure 1A, C, D). This was coupled with microthrombi in medium to small sized vessels in some patients (Figure 3F). Fibrin thrombi mostly located in the subpleural region were also noted. There were also foci of fibrosis in the lungs (Case 19). The significant findings within the lungs are shown in Figures 1 and 2 and 3B.

Other organs such as the heart in Case 1 showed thickened myocytes and a thrombus within a coronary vessel but without evidence of an infarct (Figure 3A). There was also microscopic evidence of atherosclerotic narrowing with and without infarction of the myocardium in some patients. A thrombosed vessel within a myxoma (Figure 3C) in addition to a thrombus on the surface of the endocardium (Figure 3E) was seen in Case 12. Microthrombi were also noted in some glomeruli (Figure 3D), as observed in Case 10. In one patient there was widespread microthrombi in different organs, even within vasa vasora of vessels (Case 9) (Figure 3 B).

Significant histological findings in the liver included severe macrovesicular steatosis of the hepatocytes with moderate mononuclear inflammatory cell infiltrates in the portal tracts.

## Discussion

Our experience revealed that prior to autopsy, most bodies were sub-optimally preserved. This was due to delays in removing bodies to the autopsy suite because there were protocols in place that needed to be followed in order to avoid spread of the infection. The very warm, humid environmental conditions at that time of the year also contributed to the rapid deterioration of some of the bodies. In addition, all the bodies needed to be sprayed with chlorine as a disinfection practice stipulated by the accepted protocols for managing deaths from COVID-19. In order to obtain the best results from histopathological examination however, bodies and thus internal organs needed to be well preserved. It therefore took between 9 and18 hours before the bodies were put into the fridge. Then again, the autopsies had to be done in a timely manner while observing laid down autopsy protocols for carrying out autopsies on category-3 and 4 infectious organisms such as SARS-CoV-2. To achieve this, bodies of deceased patients had to be removed in a timely fashion to the COVID-19 theatre in the autopsy suite for examination.

In relation to category 3 and 4 infectious organisms, there is currently no working, completely well-equipped autopsy suite in Ghana for carrying out such autopsies. The structural design of our autopsy suites lacks the negative pressure systems with filters recommended by the WHO.^16^ In this regard, appropriate technology, as described in the methodology was employed in the autopsy suites and all autopsies carried out with improvised equipment in improvised theatres. A critical look at autopsy practice and infrastructure in Ghana is recommended to improve practice, research and evidence.

There was equal proportion of males and females in the cases presented. Thirteen (65%) of the cases were 55 years or older, a finding that is consistent with worldwide reports on COVID-19 deaths which consistently report deaths in older people who also tend to live with comorbidities.^2^,^5^ These reports, however, also show the vulnerability of younger people with comorbidities to the infection and subsequent death from COVID-19. Our findings are consistent with these reports with some deaths occurring in patients younger than 40 years in our series.^2^,^5^ These individuals had comorbidities. It is evident that all patients had other underlying health conditions with most being hypertensive and or diabetic (65%). This is in line with previous reports that the clinical presentation of COVID-19 infections varies from mild to severe disease with severe disease reportedly present in patients with co-morbidities.^6^ Other comorbid conditions in our series included complicated atherosclerosis (CAD), atrial myxoma, adrenal tumour, HIV and ‘pregnancy state’. Though there are other co-morbid conditions in Ghanaians such as sickle cell disease, pulmonary tuberculosis, there is currently no local report of pathological findings in such patients who die of COVID-19.

Forty percent (6/15) of our cases that had the nasopharyngeal swab test before death had negative results for COVID-19 but had positive results at post-mortem. This is consistent with earlier reports that broncho-alveolar lavage specimen is more sensitive than the nasopharyngeal swab.^15^,^17^ It is therefore recommended that postmortems should be encouraged in presumed COVID-19 patients who die despite testing negative for SARS-CoV-2.

Severe COVID-19 disease is characterized histopathologically by DAD with deposition of HMs that result in diffusion-perfusion mismatch in the lung characterized clinically by Acute Respiratory Distress Syndrome (ARDS) in which there is respiratory hypoxemia that is refractory to oxygen.^7^,^8^,^9^ In the acute stage of DAD there is HM formation.^6^ All our cases had HMs deposited in the lungs. Again, there was significant pulmonary edema in all the patients. Previous reports state that interstitial widening by edema and fibroblast proliferation are prominent in the organizing stage of DAD/ARDS.^5^

Additional pathological features that have been reported in the lung include reactive hyperplasia limited to some type-II pneumocytes and patchy inflammatory changes with scattered multinucleated giant cells.^8^,^9^ These were features that were present to varying degrees in some patients. The presence and significance of multinucleated giant cells remain unexplained. In some patients, a clinical diagnosis of bronchopneumonia was made following initial clinical findings suggestive of such.^5^ These included findings on clinical examination (auscultation) and imaging.

A number of our patients had pulmonary thromboemboli within both the major pulmonary artery branches and smaller pulmonary artery branches (70%). The role of stasis, endotheliitis and other factors that affect susceptibility to thrombosis in relation to COVID-19 infection needs to be properly studied in order to understand the causal role played by COVID-19 infection. However, the prominent role of endotheliitis in COVID-19 infection related thrombosis has been reported with suggestions that infection with SARS-CoV-2 results in endotheliitis in several organs from their involvement by the virus and as part of the host inflammatory response. It is believed that a strategy that targets endotheliitis in susceptible patients is likely to improve survival in such patients.^14^,^17^ Considering most of our patients had DM and or HPT in addition to being obese and also being bed ridden because of sickness, it is likely that COVID-19 infection has a contributory role in predisposing these patients to thrombosis.

Pathological features in other organs including the liver and heart were less significant with mostly a confirmation of pre-existing hypertensive heart disease or fatty liver as in other reports.^6^,^17^ The finding of fatty liver in some patients may be related to their preexisting obesity as part of a metabolic syndrome and not to the COVID-19 infection. Again, CLVH is an end result of systemic hypertension. In our experience, the causal role of COVID-19 infection in the context of pre-existing chronic disease or infection is difficult to unravel.

## Conclusion

The majority of the patients autopsied had pre-existing medical conditions with the commonest being HPT and DM. The most significant pathological feature of the patients who died of COVID-19 was DAD. A management approach that recognizes early onset ARDS/DAD and aggressively treats or prevents further damage to the lung may improve the survival of patients.
